# Subclinical Hypothyroidism as the Most Common Thyroid Dysfunction Status in Children With Down’s Syndrome

**DOI:** 10.3389/fendo.2021.782865

**Published:** 2022-01-04

**Authors:** Kamila Szeliga, Aleksandra Antosz, Karolina Skrzynska, Barbara Kalina-Faska, Aleksandra Januszek-Trzciakowska, Aneta Gawlik

**Affiliations:** ^1^Department of Pediatrics and Pediatric Endocrinology, Faculty of Medical Science, Medical University of Silesia, Katowice, Poland; ^2^Department of Pediatrics and Pediatric Endocrinology, Upper Silesian Medical Center in Katowice, Katowice, Poland; ^3^Endocrinological Outpatient Clinic, Upper Silesian Medical Center in Katowice, Katowice, Poland

**Keywords:** subclinical hypothyroidism, children, trisomy 21, levothyroxine, treatment

## Abstract

**Introduction:**

Thyroid dysfunctions are one of the most common abnormalities coexisting in children with Down’s syndrome (DS) and have been reported in up to 54% of cases.

**Aim of the Study:**

The purposes of this retrospective study were to investigate the course of subclinical hypothyroidism in children with DS, to evaluate the thyroid function of these subjects in relation to the risk of developing overt thyroid disease and autoimmunity, and to identify clinical and biochemical characteristics of patients prescribed L-T4 therapy in children and adolescents with DS and SH.

**Material and Methods:**

The records of DS patients referred to the Endocrinology Outpatient Clinic between 2010 and 2015 for screening of thyroid function were observed till the end of 2019 June and analyzed retrospectively. The children diagnosed with congenital hypothyroidism, acute lymphoblastic leukemia, and seizures and treated with drugs that may have interfered with thyroid function like lithium, antiepileptic, or iodinated drugs and glucocorticoids were excluded from the study.

**Results:**

The data of 77 DS patients were collected, evaluated, and analyzed. The study group consisted of 73 patients (32 girls and 41 boys with the mean age at baseline of 3.0 ± 4.5 years). A total of 63/73 (87%) children were diagnosed with SH. The 16/63 (25.4%) patients were followed-up without the treatment (group SH-T0), and therapy with levothyroxine (L-T4) was introduced in 47/63 (74.6%) SH children with a mean dosage of 1.8 ± 1.0 μg/kg/day (group SH-T1). Thyroxine supplementation did not improve growth expressed as ΔhSDS (0.1 ± 1.3, ranged −2.1 to 3.8 in SH-T0 vs. 0.0 ± 0.7, ranged −1.7 to 1.4 in SH-T1, *p* = 0.96) and ΔBMI Z-score (0.3 ± 0.9, ranged −0.9 to 2.6 in SH-T0 vs. 0.3 ± 1.1, ranged −2.1 to 2.9 in SH-T1, *p* = 0.65). Positive anti-TPO and anti-TG antibodies were detected in 7/63 (11.1%) DS cases.

**Conclusions:**

SH is the most frequent presentation of thyroid gland dysfunction in DS children. A small percentage of patients develop an overt hypothyroidism, particularly in females with mostly positive titer of antithyroid autoantibodies.

## Introduction

The Down’s syndrome (DS) is the most commonly recognized genetic cause of intellectual disability (1) with a total prevalence 10.41 per 10,000 live births in 2016 according to the population-based EUROCAT registries (2). It is proven that the extra genetic material from chromosome 21 results in having multiple malformations and medical conditions that are higher than in the general population (1), which have a negative impact either on life expectancy or quality of life (3). In addition to the increased risk of hearing loss (75%), obstructive sleep apnea (50%–79%), congenital heart defects (CHD) (50%), and neurologic dysfunction (1%–13%) in children with DS have a higher rate of endocrine disturbances (4).

Thyroid dysfunctions are one of the most common abnormalities coexisting in DS children and have been reported in up to 28%–40% of the patients with the frequency increasing with age up to 54% (5–7).

Subclinical hypothyroidism (SH) is a particularly frequent thyroid dysfunction, being reported between 25% and 32% (8, 9) and up to 50% and 60% of patients with DS (10). The diagnosis of SH is made when serum thyroid-stimulating hormone (TSH) is mildly raised with the levels of total or free thyroxine (fT4) and triiodothyronine (fT3) within their reference ranges (11). It is suspected that in the infants and young children with DS, the subclinical thyroid dysfunction is caused by the delay in the maturation of the hypothalamic-pituitary-thyroid axis (HPT) and is probably a transitory and self-limiting process not requiring treatment (12, 13). Different hypotheses are the cutdown of the dopaminergic tonus both on the hypothalamus and the pituitary gland, which causes an increased secretion of TSH. That, sequentially, if associated with the downregulation of thyroid receptors of TSH, provides normal baseline values of thyroid hormones. Receptor resistance is also suspected (14).

Patients suffering from DS are well known to have an increased prevalence of autoimmune disorders affecting both endocrine and nonendocrine organs (15). Previous studies have suggested that DS is strongly associated with Hashimoto’s thyroiditis (HT) and positive titer of thyroid autoantibodies are found in 13%–34% of DS patients (16).

By contrast to healthy pediatric controls, HT is diagnosed earlier (mean age: 6.5 years) without gender predominance and with higher prevalence of extra-thyroidal autoimmune disorders (15, 17), such as Addison’s disease, allergic dermatitis, coeliac disease, diabetes mellitus, hypo- and hyperthyroidism, autoimmune hemolytic anemia, dermatomyositis, multiple sclerosis, pernicious anemia, polyarteritis nodosa, rheumatoid arthritis, scleroderma, Sjogren’s syndrome, and systemic lupus erythematosus (18). The incidence of family history for thyroid disturbance is lower. In addition, the most common hormonal pattern in HT is SH, followed by overt hypothyroidism, whereas euthyroid profile is rare (19). A higher baseline TSH levels and underlying HT in SH children predisposes to worsening of thyroid status over time (20).

The deterioration of thyroid function to Grave’s disease during observation period is more common in patients with SH and HT (25%) as well as there is a higher risk of developing overt hypothyroidism (21).

Furthermore, there is a controversy regarding treatment of subclinical hypothyroidism given unclear benefits (22). Undoubtedly, it is recommended in patients with Down’s syndrome to have their thyroid function regularly monitored (23).

The purpose of the retrospective study was to investigate the course of subclinical hypothyroidism diagnosed in children with DS, to monitor the thyroid function in the context of autoimmune and overt thyroid disorder risk as well as the rationale for prescribing the l-thyroxine to young patients with DS and SH.

## Material and Methods

The records of all the patients with genetically confirmed DS and referred to the Endocrinology Outpatient Clinic of the Medical University of Silesia between 2010 and 2015 for screening of thyroid function observed till the end of 2019 June were analyzed retrospectively. The study was performed in a noniodine-deficient area. We excluded from the study the children who were treated with drugs that may have interfered with thyroid function like lithium, antiepileptic drugs, glucocorticoids, or iodinated drugs, as well as the patients who were diagnosed with acute lymphoblastic leukemia and seizures.

### Ethical Considerations

The study was approved by the Ethics Committee of the Medical University of Silesia.

The patients’ rights were also approved according to the Declaration of Helsinki. Informed consent was obtained from each patient over the age of 16, a parent, or a legal custodian.

### Clinical and Biochemical Measurements

The following data were collected: gender and age at the first visit in outpatient clinic; auxological data: height and weight, serum concentrations of thyroid-stimulating hormone (TSH), free thyroxine (fT4), and free triiodothyronine (fT3); and titer of antithyroid autoantibodies: antiperoxidase (anti-TPO) and antithyroglobulin (anti-TG), family history, clinical symptoms suggesting thyroid disease, and the duration of the follow-up at the Outpatient Clinic. Clinical condition, patients’ complaints, as well as anthropometric data were collected at a regular physical examination every 3 to 6 months or anamnesis during the visit. A standard stadiometer was used to measure the patients’ height. Height, weight, and BMI percentiles (pc) were evaluated according to specific growth charts dedicated to children with DS (24). Height standard deviation score (HSDS) was calculated from population standards for children (24) using the following formula: hSDS = child’s height − height for 50 pc/0.5 * (height 50 pc − height 3 pc). Body mass index (BMI) was expressed as kilograms per square meter and assessed in regard to percentile charts for BMI (25). The BMI Z-score was calculated using online calculator provided by Baylor College of Medicine (26) and used to compare group of patients and to assess changes in body weight during follow-up. Following the WHO standards, cutoffs for childhood overweight and obesity were >+1 SDS which corresponds to 85th pc for “overweight” and >+2SDS which corresponds to 95th pc for obesity (27, 28).

A chemiluminescent immunometric assay (Siemens, Immulite 2000 Free T4, Immulite 2000 Third Generation TSH, Malvern, PA, USA) was used to detect the serum concentrations of TSH, fT4, and fT3. The concentrations of antithyroid peroxidase antibody (anti-TPO Abs) and autoantibodies to thyroglobulin (anti-TG Abs) were determined with enzyme-labeled, chemiluminescent-sequential immunometric assay (Siemens, Immulite 2000 anti-TPO Ab, Immulite 2000 Anti-TG Ab, USA). The range between 11.5 and 22.7 pmol/l (0.8–1.9 ng/dl) was considered normal for fT4. Anti-TG Abs values >40 IU/ml and anti-TPO Abs titer >35 IU/ml were defined as positive. Thyroid ultrasound examination was performed at least once during the follow-up period, using Acuson Antares (Siemens Medical Solution USA, Inc.) with a VFX 13-5 linear transducer. A thyroid autoimmune disorder (Hashimoto’s thyroiditis) was considered when the diffused low echogenicity was detected in the presence of anti-TPO Abs and/or anti-TG Abs, with different presentation patterns: subclinical or overt hypothyroidism, euthyroidism, more rarely subclinical, or overt hyperthyroidism.

With regard to the thyroid function during the observational period, patients with DS were evaluated according to the TSH and fT4 serum levels and classified into three groups: (i) euthyroidism (EuT), diagnosed when the values of fT4 and TSH were within their reference ranges; (ii) subclinical hypothyroidism (SH), diagnosed in children with no evident clinical signs of hypothyroidism, if TSH during the follow-up period was elevated above 4.0 mIU/ml and fT4 remained within the reference range; and (iii) overt hypothyroidism (OH), diagnosed when TSH was higher than 10.0 mIU/ml and fT4 was below the lower limit of the reference range. Thyroid autoimmunity was documented by the presence of thyroid autoantibodies (anti-TPO Abs and/or anti-TG Abs).

Patients who were observed over a period of time without treatment were assigned to group SH-T0 and patients who started treatment with levothyroxine (L-T4) to group SH-T1.

A dose of L-T4 was expressed as an average dosage, using the recommended amount of L-T4 on the first and last recorded visit in relation to body weight and was presented as micrograms per kilogram per day.

### Statistical Analysis

Calculation was made using R programming language (R Core Team, 2008) working in RStudio environment (29).

Numerical data were expressed as mean ± SD or median and range values, as appropriate. Categorical variables are frequency and percentage.

The Shapiro-Wilk test was used to check whether or not a continuous variable follows a normal distribution.

Comparison between groups was performed using Student’s *t*-test for normally distributed data (age at baseline, age at the end of FU, length of FU, hSDS at baseline, hSDS at the end of FU, BMI Z-score at baseline, BMI Z-score at end of FU, TSH, and fT4 at baseline and end of FU), and Wilcoxon test was dedicated to nonnormally distributed data (clinical outcomes: ΔhSDS and ΔBMI Z-score).

Correlation analysis was performed using Spearman’s correlation coefficient for nonnormally distributed data. *p*-value <0.05 was considered statistically significant.

## Results

The data of 77 patients with suspected thyroid disorders were collected, evaluated, and analyzed.

**Figure 1:**
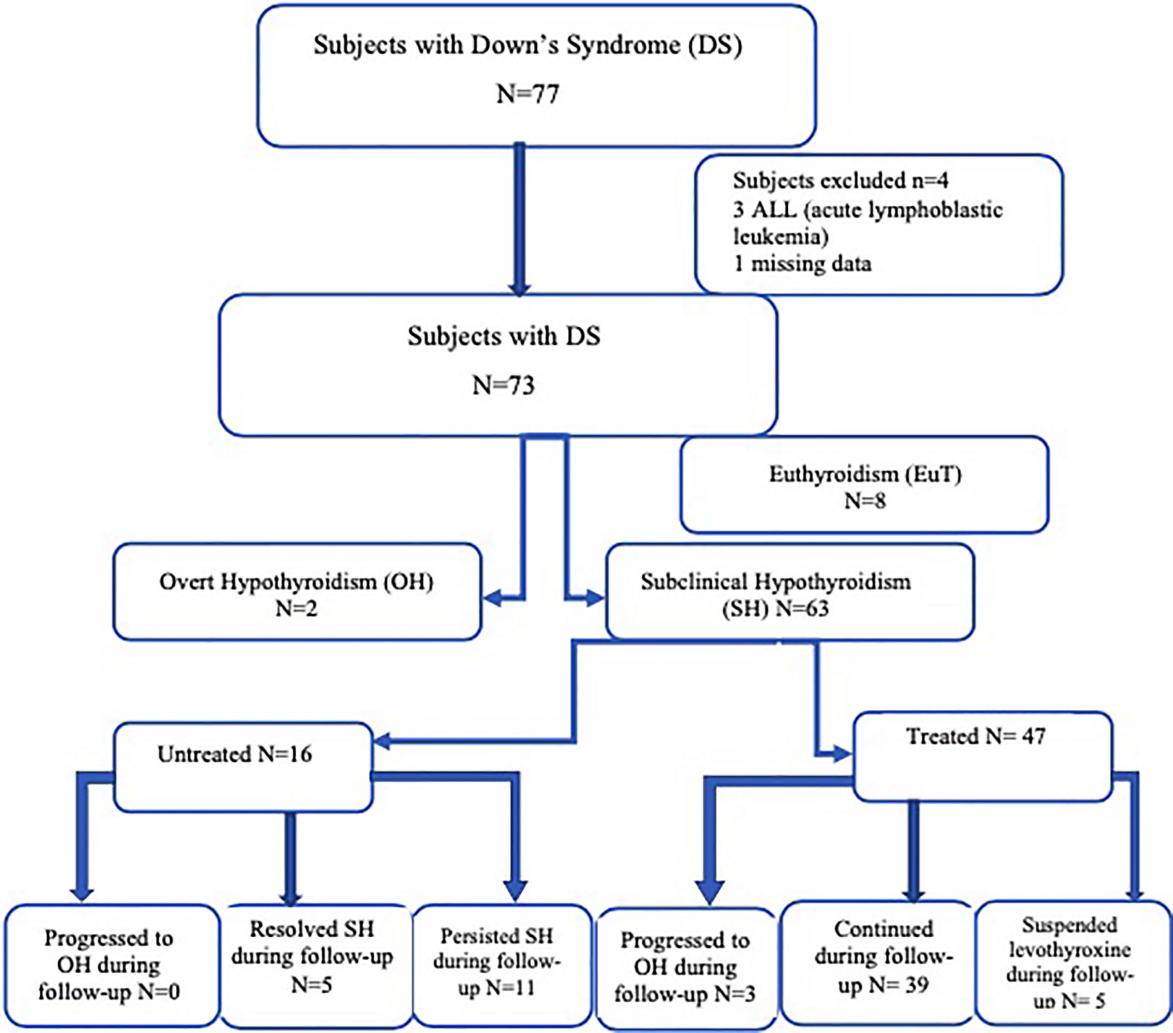
The thyroid function patterns of the DS subjects.

**Figure 1** presents the thyroid function patterns of the DS subjects.

Four patients were excluded from the study: 3 children diagnosed and treated because of acute lymphoblastic leukemia and 1 patient was lost to follow-up. The group consisted of 73 patients (32 girls and 41 boys with the mean age at the first visit of 3.0 ± 4.5 years (range from 0.1 to 16.9 years, median 0.8 years).

Subclinical hypothyroidism (SH) was the most common manifestation of thyroid gland disturbance (*n* = 63/73, 38 boys, 25 girls). In 2/73 (2.7%) females, overt hypothyroidism (OH) was diagnosed at baseline, whereas 8/73 (10.9%) DS individuals had euthyroid profile (EuT). In this time frame, neither presented with hyperthyroidism nor had congenital hypothyroidism. The positive family history towards thyroid gland disease was detected in one case from SH group.

The children from the study group were averagely observed for 44.9 ± 29.1 months (range: 2.4–106.2 months, median was 47.8 months) and in general had 8 clinical reviews.

The detailed characteristic of patients from group SH (SH-T0 and SH-T1) as well as from group EuT is presented in **Table 1**.

**Table 1:** Clinical and biochemical characteristic of patients.

	Variable unit	SH				EuT (*n* = 8) mean ± SD (range)
		General (*n* = 63) mean ± SD (range)	SH-T1 (*n* = 47) mean ± SD (range)	SH-T0 (*n* = 16) mean ± SD (range)	*p* (SH-T1) and SH-T0)	
**Clinical phenotypes of patients**	**Sex (F/M)**	25/38	20/27	5/11	–	5/3
	**Age at the baseline (years)**	3.0 ± 4.5 (0.1–16.9)	3.5 ± 4.9 (0.1–16.9)	1.4 ± 2.6 (0.1–8.7)	<0.05 (*t* = 2.17)	6.1 ± 5.9 (0.4–14.8)
	**Age at the end of FU (years)**	6.3 ± 4.8 (0.1–17.9)	7.1 ± 5.0 (0.1–17.9)	4.2 ± 3.3 (0.8–12.2)	<0.05 (*t* = 2.63)	9.5 ± 5.5 (3.6–7.2)
	**Length of FU (months)**	44.9 ± 29.1 (2.4–106.2)	48.5 ± 31.1 (2.4–106.2)	34.8 ± 20.2 (9.0–66.3)	<0.05 (*t* = 2.02)	45.8 ± 20.7 (12.3–70.7)
	**hSDS at the baseline**	−0.1 ± 1.3 (−5.8 to 2.7)	0.0 ± 1.1 (−3.0 to 2.7)	−0.3 ± 1.8 (−5.8 to 1.5)	0.28 (*t* = 1.09)	−0.5 ± 0.9 (−1.5 to 1.4)
	**hSDS at the end of FU**	−0.1 ± 1.1 (−3.8 to 2.7)	0.0 ± 1.2 (−3.8 to 2.7)	−0.2 ± 0.9 (1.9–1.3)	0.67 (*t* = 04.15)	−0.2 ± 0.9 (−1.1 to 1.4)
	**BMI <3rd pc/>85th pc**	5/7	5/6	0/1	–	2/3
	**BMI Z-score at the baseline**	0.2 ± 1.4 (−3.7 to 2.4)	0.3 ± 1.4 (−3.7 to 2.4)	−0.1 ± 1.5 (−3.6 to 2.9)	0.42 (*t* = 0.841)	−0.1 ± 2.6 (−4.1 to2.8)
	**BMI Z-score at the end**	0.5 ± 1.3 (−5.8 to 2.4)	0.6 ± 1.4 (−5.8 to 2.4)	0.2 ± 1.0 (−0.9 to 2.1)	0.41 (*t* = 0.816)	−0.2 ± 2.1 (−4.2 to 2.3)
	**Hashimoto’s thyroiditis during FU**	7/63 (11.1%)	6/47 (12.8%)	1/16 (6.3%)	–	0/8 (0%)
**Biochemical phenotype**	**Dosage of L-T4 (µg/kg/day)**	1.8 ± 1.0 (0.3–6.6)	1.8 ± 1.0 (0.3–6.6)	0	–	0
	**TSH (mIU/ml) at the baseline**	6.7 ± 4.6 (1.3–23.6)	6.8 ± 5.2 (1.3–23.6)	6.4 ± 2.3 (4.1–10.4)	0.67 (*t* = 0.425)	2.2 ± 0.6 (1.4–3.1)
	**TSH (mIU/ml) at the end of FU**	4.5 ± 2.2 (0.5–12.5)	4.6 ± 2.5 (0.5–12.5)	4.3 ± 1.3 (2.3–7.7)	0.45 (*t* = 0.745)	2.7 ± 0.7 (1.4–3.5)
	**fT4 (ng/dl) at the baseline**	1.4 ± 0.3 (0.8–2.1)	1.4 ± 0.3 (0.8–2.1)	1.5 ± 0.3 (0.8–2.1)	0.79 (*t* = 0.221)	1.3 ± 0.2 (1.1–1.6)
	**fT4 (ng/dl) at the end of FU**	1.3 ± 0.3 (0.8–2.0)	1.3 ± 0.3 (0.8–2.0)	1.3 ± 0.2 (1.0–1.7)	0.69 (*t* = 0.39)	1.3 ± 0.2 (1.1–1.7)
	**Diagnosis at the end of FU**	SH—55^*5^/63	SH—44^*4^/47	SH—11^*1^/16	–	SH—1/8
		EuT—5/63	EuT—0/47	EuT—5/16		EuT—7/8
		OH—3^*2^/63	OH—3^*2^/47	OH—0/16		OH—0/8

t, test statistic (Student’s t-test); SH, subclinical hypothyroidism; OH, overt hypothyroidism; EuT, euthyroid; SD, standard deviation; SH-T1, patients from group SH with levothyroxine treatment; SH-T0, patients from group SH without treatment; EuT, euthyroid patients; F, female; M, male; FU, follow-up; hSDS, height standard deviation score; BMI, body mass index; Pc, percentile; CHD, congenital heart disease; L-T4, levothyroxine; TSH, thyroid-stimulating hormone; fT4, free thyroxine; Abs, antithyroid autoantibodies.

^*^Children with (+) Abs.

In 49/63 (77.8%) DS patients with SH, serum TSH concentrations were between 4.0 and 10.0 mIU/ml, whereas in 14/63 (22.2%) cases, the TSH level was higher than 10 ± 4.0 mIU/ml (range: 10.1–23.6 mIU/ml, median: 12.5 mIU/ml).

Positive anti-TPO and anti-TG antibodies during follow-up were detected in 7/63 (11.1%) cases, in 6 girls and 1 boy at a mean age 6.8 ± 5.0 years (range 1.4–13.2 years, median 5 years) (**Table 1**).

**Figure 2:**
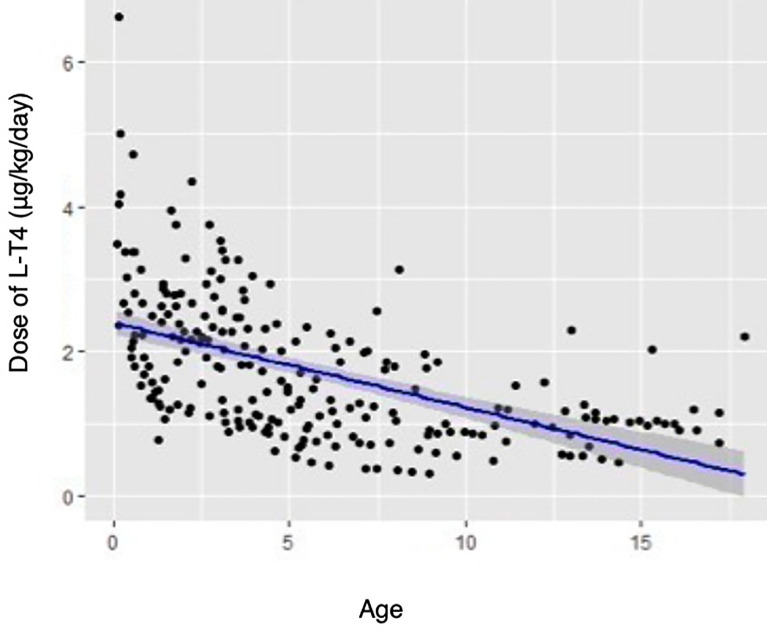
The relation between doses of L-T4 and age in patients with SH.

The supplementation treatment with L-T4 was introduced in 47/63 (74.6%) SH children with a mean dosage of 1.8 ± 1.0 μg/kg/day (ranged from 0.3 to 6.6 μg/kg/day, median 1.6 μg/kg/day). It was more often applied in older children (**Table 1**). The younger children, the dose per kilograms was higher (Rho-Spearman analysis, Rs −0.628, *p* < 0.001—**Figure 2**). Patients with and without CHD required a similar doses of L-T4 (*p* = 0.26). The therapy with L-T4 did not influence on growth expressed as ΔhSDS (0.1 ± 1.3, ranged −2.1 to 3.8 in SH-T0 vs. 0.0 ± 0.7, ranged −1.7 to 1.4 in SH-T1, *p* = 0.96) and on ΔBMI Z-score (0.3 ± 0.9, ranged −0.9 to 2.6 in SH-T0 vs. 0.3 ± 1.1, ranged −2.1 to 2.9 in SH-T1, *p* = 0.65).

CHD was found in 21/63 (33.3%) DS patients. The frequency of CHD was similar in both groups of patients: SH-T0 (6/16, 37.5%) and SH-T1 (15/47, 31.9%) (*p* = 0.5). Within the observational period, HT was diagnosed in 6/47 (12.8%) patients from group SH-T1: in five girls and one boy. One girl had a history of other autoimmune disorders: celiac disease and diabetes mellitus type 1. HT patients averagely received 1.9 ± 0.8 μg/kg/day of L-T4, median 1.8 μg/kg/day, ranging from 0.3 to 4.4 μg/kg/day.

The therapy with L-T4 was suspended in 5/47 (10.6%) cases. the TSH level was normalized <4 mIU/ml in 2/47 (4.3%) patients; whereas, hyperthyrotropinemia was still observed in 3/47 (6.4%) subjects.

There were no differences with regard to TSH and fT4 levels, BMI *Z*-score, hSDS Z-score at the baseline, as well as presence of CHD between patients who underwent therapy with L-T4 and those who did not.

In the SH-T0 group, SH resolved spontaneously in 5 of 16 cases and there was no progression to overt hypothyroidism.

The clinical and biochemical presentation of 2 female patients who were diagnosed on the baseline with overt hypothyroidism (OH—Girl I and Girl Il) as well as 3 girls who developed OH over time was included in **Table 2**. In our retrospective study, we did not find any male patients who met the criteria of OH.

**Table 2:** Children with overt hypothyroidism (OH) characteristics.

	OH at baseline		SH patients (F) who developed OH		
	Girl I	Girl II	Girl III	Girl IV	Girl V
**Age at baseline (years)**	15.2	10.2	14.3	0.8	0.8
**Age at end of FU (years)**	17.9	16.3	14.8	5.2	5.2
**Length of FU (months)**	32.8	73.5	5.2	52.0	52.1
**TSH (mIU/m)] at baseline**	77.8	5.9	10.5	10.0	10.0
**TSH (mIU/ml) at end of FU**	13.1	1.9	5.34	4.1	5.0
**fT4 (ng/dl) at baseline**	0.7	0.79	0.84	1.45	1.54
**fT4 (ng/dl) at end of FU**	1.0	1.7	0.78	0.78	0.76
**Presence of Abs**	1	1	0	1	1
**CHD**	1	0	0	1	0
**hSDS at baseline**	−1.4	1.9	2.4	0.3	0.0
**hSDS at end of FU**	−1.3	1.5	2.7	0.9	0.9
**BMI (kg/m^2^)**	18.9	24.8	27.7	15.5	14.3
**Dosage of L-T4 (µg/kg/day)**	1.6	1.1	0.5	2.7	2.5
**Other autoimmune diseases**	–	–	AA, AD	DMt1	–
**Diagnosis at end of FU**	OH	OH	OH	OH	OH

SH, subclinical hypothyroidism; OH, overt hypothyroidism; EuT, euthyroid patients; SH-T1, patients from group SH with levothyroxine treatment; SH-T0, patients from group SH without treatment; F, female; M, male; FU, follow-up; SD, standard deviation; TSH, thyroid-stimulating hormone; fT4, free thyroxine; Abs, antithyroid autoantibodies; CHD, congenital heart disease; hSDS, height standard deviation score; BMI, body mass index; L-T4, levothyroxine; AA, alopecia aerata; AD, atopic dermatitis; DMt1, diabetes type 1; CD, coeliac disease.

Clinical symptoms of DS patients during follow-up are presented in **Table 3**.

**Table 3:** Clinical symptoms in DS patients.

	EuT (*n* = 8)	SH-T1 (*n* = 47)	SH-T0 (*n* = 16)	OH (*n* = 2)
Overweight or obesity (*n* (%))	3 (37.5)	6 (8.5)	1 (6.3)	1 (50)
Underweight (*n* (%))	2 (25.0)	5 (10.6)	0 (0)	0 (0)
Hypotonia (*n* (%))	2 (25.0)	8 (17.0)	3(18.8)	0 (0)
Dry skin (*n* (%))	0 (0)	7 (14.9)	0 (0)	0 (0)
Dry hair (*n* (%))	0 (0)	1 (2.1)	0 (0)	0 (0)
Goiter (*n* (%))	0 (0)	1 (2.1)	0 (0)	1 (50)

DS, Down’s syndrome; EuT, euthyroid patients; SH-T1, patients with subclinical hypothyroidism with levothyroxine treatment; SH-T0, patients with subclinical hypothyroidism without treatment; OH, overt hypothyroidism.

## Discussion

### Prevalence of Thyroid Diseases in Children With DS

In subjects with DS, the prevalence of SH is higher than in the general healthy pediatric population and has been variously estimated at 25.3% (9), 18.6% (17), 19.6% (30), 39% (31), and 48.9% (32) to 60% (10), whereas the congenital hypothyroidism, autoimmune thyroiditis and hyperthyroidism are found in 4.0%–6.1% (5, 31, 33), 7.6%–31% (15, 34–37), and 0.65%–3% (5, 9, 34), respectively.

Among 77 patients referred for endocrinological observation, the SH was diagnosed in vast majority of patients (63/77 DS cases—81.8%), which is one of the highest percentages reported in the literature. Such high frequency of SH might result from our Polish diagnostic criteria of SH in pediatric population when it is compared with other publications (16, 17, 34). The most common cause of SH in our DS population was nonautoimmune thyroid disorder (88.9%), in accordance with the literature (6, 38–42).

### Clinical Symptoms Suggesting Hypothyroidism

As proclaimed by definition of subclinical condition, it is “without symptoms,” patients with SH should be asymptomatic or the symptoms of thyroid dysfunction may be minimal or nonspecific (43). DS is a clinical condition which requires a multidisciplinary care and the potential symptoms of hypothyroidism are easy to overlook, however distinguishing physical symptoms and signs suggesting hypothyroidism from similar ones present in children with DS is a common difficulty (that is: decreased physical activity, feeding difficulties, hypotonia, macroglossia, developmental delays, weight gain, constipation) (44). In comparison with the patients described by Such et al. (45) in which 80% of SH children and adolescents presented at least one symptom suggesting hypothyroidism, we found that in almost half of the DS cases with SH clinical manifestation of hypothyroidism was detected.

In addition, symptoms of hypothyroidism are nonspecific because they overlap with those inherent to DS so it seems reasonable to rely on TSH and free thyroid hormone levels rather than on the symptoms.

### Hashimoto’s Thyroiditis

It is known that genetic defect in DS triggers the immune system leading to many autoimmune disorders including HT, which is the most common autoimmune disease in this group of patients (15). Furthermore, the risk of progression to overt hypothyroidism over time is higher in the DS children with HT-related SH (46). In our study, HT during follow-up was diagnosed in 7/63 (11.1%) DS individuals. No positive family history of autoimmune thyroid diseases in those groups of patients was identified. According to Karlsson et al. (16), thyroid autoantibodies are found in 13%–34% of DS patients, which consisted our cohort. In their prospective evaluation, autoimmune thyroid disease occurred commonly after the age of 8 years, while the prevalence in preschool children was uncommon. In our group, the onset of HT majorly took place before the 7th birthday.

Notwithstanding, in another original printing (47), it was found that DS patients with elevated TSH concentration had low risk of progression to Hashimoto’s thyroiditis—10% for males and 6% for females. Similar conclusions that Hashimoto’s thyroiditis in DS children occurs without female predominance were drawn by Aversa et al. (17). In our study, the prevalence of Hashimoto’s thyroiditis among children from group SH was 9.5% for female and 1.6% for male.

We did not observe in our study group a shifting between Hashimoto’s thyroiditis and Grave’s disease which happened in literature and was described in previous articles with frequency of 7.1%–8.1% of DS patients (17, 19).

### Congenital Heart Disease and Thyroid Dysfunction

The results of our study show that the congenital heart disease (CHD) was found in almost one-third of the DS children, which is consistent with the other studies (11). Mıhçı et al. analyzed retrospectively 187 cases of the children with DS (1993–2005) and found that 72.73% DS patients had CHD, and there was no statistical relationship between having any kind of CHD and level of TSH and fT4 (48).

Some investigators suggest that the therapy with levothyroxine should be recommended in children with DS, even in mild cases of SH, because impairment of the cardiac function might worsen their clinical condition and thus influence their quality of life and life expectancy.

The congenital heart disease was diagnosed in both groups (SH-T0 and SH-T1) with similar frequency, which suggested that the presence of CHD was not an indicator for administrating the L-T4.

### Treatment of SH With L-T4

Nowadays, it is uncertain if SH should be treated in DS children at all, at what level of TSH the L-T4 therapy should be applied, or whether levothyroxine has any potential benefits in intellectual development, puberty, as well as growth.

According the literature, the diagnosis of Hashimoto’s thyroiditis and/or other autoimmune diseases, high TSH levels (>9 mIU/ml) (41) and the presence of subclinical hypothyroidism in the context of a genetic syndromes (DS or Turner’s Syndrome) are some of the indications for undertaking treatment with L-T4 (41, 49). The treatment with L-T4 might be also considered in patients with goiter and signs of hypothyroidism and/or proatherogenic metabolic abnormalities (50). There was lack of statistical differences between groups (SH-T0 vs. SH-T1); however, it seems the most probable that the above characteristics of patients have influenced on the decision on starting the treatment with LT-4.

According to our data, the treatment of L-T4 was introduced in vast majority of DS patients with subclinical hypothyroidism (47/63%–74.6%). Eighty-six percent of patients diagnosed with Hashimoto’s thyroiditis were treated with L-T4, in similar to patients from a large study conducted by Iughetti (39), in which almost all patients with antithyroid autoantibodies required L-T4. Nevertheless, there are a few studies systematically examining the frequency of thyroid disease in children with DS and the effects of l-thyroxine treatment in subjects with DS and SH.

In our study, we found no differences between TSH and fT4 serum concentrations between both SH-T0 and SH-T1 groups of patients. Additionally, there was no differences between patients from both groups when taking into account hSDS, BMI Z-score, and frequency of congenital heart disease. It is still a matter of debate whether DS patients with subclinical hypothyroidism may benefit from substitution therapy with L-T4. On the other hand, the consequences of longstanding observation are also uncertain and there is lack of studies which may solve that issue. Our data put in evidence that levothyroxine therapy has no positive influence on height and BMI Z-score at the end of the follow-up. There are a few publications on L-T4 treatment in children with DS and SH with opposing results (a small positive effect on growth and body weight vs. no effect) (3, 16, 51, 52). However, some authors report that (53–55) l-thyroxine replacement in the first 2 years of life improves the growth in young infants with DS. Furthermore, we observed in our study a wide variety of dosage of L-T4 sufficient to maintain TSH within their reference range (1.8 μg/kg/day, 0.3–6.6 μg/kg/day), which might be explained by the fact that our group consisted of children in different ages. Moreover, in DS children, the recommended doses of L-T4 are higher in comparison with healthy individuals with SH (45). According to the study presented by Kowalczyk et al. (51), the mean dosage of L-T4 administrated for children aged 0–2 years was 1.51 ± 1.03, while for patients in age of 2–4 and >4, the mean dosage were 1.86 ± 0.82 and 2.34 ± 0.82 μg/kg/day, respectively. Those data show that there is still no established definitive recommended dose of L-T4 which may protect patients from developing overt hypothyroidism as well as is enough to sustain the normal TSH serum concentration in general healthy children as well as in patients with chromosomopathies. Moreover, malabsorption is quite a common condition in DS and individuals with DS have more than 18 times the incidence rate of CD compared with the general population, which may necessitate the use of higher doses of L-T4 (55).

Children taken into consideration were observed and longitudinally assessed for 4 years on average and had approximately 8 endocrine reviews. During every attended review on which the blood samples were obtained took place approximately every 5 to 6 months, it seemed to be an extremely stressful procedure for the child as well as for the parent.

Therefore, the question arises whether in the case of stable disease it could be recommended to visit the endocrinologist every 12 months in order to reduce the additional stress in a chronically ill child who is under the care of many doctors. AAP recommends (56) to measure TSH annually or sooner if the child has symptoms that could be related to thyroid dysfunction.

### Detoriation of Thyroid Function

As demonstrated in article, at the end of follow-up there was a progression from SH to overt hypothyroidism in 3/63 (4.8%) patients. The group consisted of only females with TSH detected on the baseline between 10.0 and 10.5 mIU/ml, whereas the mean value of TSH in overall SH was lower (6.7 ± 4.6 mIU/ml). In addition, 2/3 of girls were diagnosed with Hashimoto’s thyroiditis and with other autoimmune disorders, such as alopecia areata, diabetes mellitus type 1, and atopic dermatitis. Literature data reported that deterioration over time is significantly influenced by thyroid autoimmunity and higher TSH values at baseline, which corresponds to prospective study published by Pepe (38). In contrary, the female dominance was not proved in a retrospective study conducted by Aversa et al. (17). Iughetti et al. (39) in their longitudinal study concluded positive titer of antithyroid autoantibodies either were associated with higher odds or were a better predictor of severe hypothyroidism.

However, due to small size group of DS patients with OH, definitive and binding conclusions for further analyses cannot be drawn.

### Limitations

The main limitation of our study is its retrospective character and absence of the control group of patients with thyroid disorders and without genetically confirmed DS. Although the collected data come from probably the largest pediatric clinical center in the southern Poland, it may not provide a representative sample from Polish patients with Down’s syndrome. Moreover, we were not able to show the potential beneficial influence of the therapy with levothyroxine on the mental and physical development besides growth and weight of our patients.

### Conclusions

Subclinical hypothyroidism is the most frequent presentation of thyroid gland dysfunction in DS children of nonautoimmune etiology. The treatment of L-T4 was introduced in vast majority of DS patients with SH. A small percentage of patients develop an overt hypothyroidism, particularly in females with mostly positive titer of antithyroid autoantibodies.

There is still a lack of strict guidelines on how often patients with DS should be screened for thyroid diseases, what TSH level is a cutoff point for administrating L-T4 therapy, and if L-T4 therapy has any positive influence on growth, physical, and mental development.

Answer to these questions may be delivered by the randomized multicenter long-term trial involving children with DS and patients without any chromosomopathies diagnosed with subclinical as well as overt thyroid deficiency.

## Data Availability Statement

The original contributions presented in the study are included in the article/supplementary material. Further inquiries can be directed to the corresponding author.

## Ethics Statement

The studies involving human participants were reviewed and approved by the Ethics Committee of the Medical University of Silesia and complied with the Declaration of Helsinki guidelines. Written informed consent to participate in this study was provided by the participants’ legal guardian/next of kin.

## Author Contributions

KSz and AG designed the study, prepared the database, and wrote the manuscript. AJ-T and BK-F monitored the patients. KSz, KSk, and AA analyzed the patient database and wrote the manuscript. All authors contributed to the article and approved the submitted version.

## Conflict of Interest

The authors declare that the research was conducted in the absence of any commercial or financial relationships that could be construed as a potential conflict of interest.

## Publisher’s Note

All claims expressed in this article are solely those of the authors and do not necessarily represent those of their affiliated organizations, or those of the publisher, the editors and the reviewers. Any product that may be evaluated in this article, or claim that may be made by its manufacturer, is not guaranteed or endorsed by the publisher.
